# Supplementary material for: The Water Insecurity Experiences (WISE) Scales are suitable for use in high-income settings: findings from cognitive interviews and nationally representative surveys
Source: Int J Equity Health. 2025 Dec 4;24:338. doi: 10.1186/s12939-025-02686-x (PMC12679753; doi:10.1186/s12939-025-02686-x)
Supplement: Supplementary file 1 — Supplementary Material 1 [file 12939_2025_2686_MOESM1_ESM.pdf]

Supplementary Table 1. Complete Individual Water Insecurity Experiences Scale item phrasing.

INDIVIDUAL WATER INSECURITY EXPERIENCES (IWISE) SCALE

Items & guidance on their administration & scoring

To be read aloud if recall period is 4 weeks:
I will now ask you about your experiences with water. For each experience, we want to know how frequently this happened to you in the previous 4 weeks. Responses are never (0 days), rarely (1–2 days), sometimes (3–10 days), often (11-20 days), and always (more than 20 days).

To be read aloud if recall period is 1 year:
I will now ask you about your experiences with water. For each experience, we want to know how frequently this happened to you in the previous 12 months. Even if it happened just once during a month, we'd like you to count that month. Responses are never (0 times), rarely (in 1–2 months), sometimes (in some but not every month), and often/always (in almost every month/every month).

Abbreviation	Full phrasing	Score
**Worry**	In the last [4 weeks/1 year] how often did you worry that you would not have enough water for all of your needs?	
**Interruption**	How often has your main water source been interrupted or limited in any way in the last [4 weeks/1 year]?	
**Clothes**	How often have problems with water meant that your clothes could not be washed?	
**Plans**	How often did you have to change schedules or plans because of problems with water?	
**Food**	Still thinking about the last [4 weeks/1 year], how often did you change what you ate because of problems with water?	
**Hands**	How often were you not able to wash your hands after dirty activities because of problems with water?	
**Body**	How often were you not able to wash your body because of problems with water?	
**Drink**	How often did you not have as much water to drink as you would have liked?	
**Anger**	Still thinking about the [last 4 weeks/1 year], how often did you feel angry because of problems you were experiencing with water?	
**Sleep**	How often did you go to sleep thirsty because there was no water to drink?	
**No Water**	How often did you have no usable or drinkable water whatsoever?	
**Shame**	How often did you feel shame because of problems you were experiencing with water during the last [4 weeks/1 year]?	
	**Total**	

Notes:

1. The interviewer should repeat the scale responses as necessary after the first item. Although the respondents are reminded of the timeframe in items 1, 2, 5, 9 and 12, the interviewer should repeat the time frame or responses only as frequently as necessary.
2. See additional guidance, including on item ordering, probes, item adaptation, and scoring in the WISE Scales Manual: https://doi.org/10.21985/n2-20sc-f113 and www.WISEscales.org.
3. WISE Scale scores are calculated by summing responses to each question. The responses to each item are scored from 0-3. For a recall period of 4 weeks, "never" is scored as 0, "rarely" as 1, "sometimes" as 2, and "often" and "always" are scored as 3. For a one-year recall period "never" is scored as 0, "in 1 or 2 months" is scored as 1, "in some but not every month" is scored as 2 and "in almost every month" and "every month" is scored as 3. The overall scores for the HWISE and IWISE Scales range from 0-36 (12x3=36). Higher scores indicate greater water insecurity.
4. IWISE Scale citation: Young, Sera L., Hilary J. Bethancourt, Zacchary R. Ritter, and Edward A. Frongillo. 2021. "The Individual Water Insecurity Experiences (IWISE) Scale: Reliability, Equivalence and Validity of an Individual-Level Measure of Water Security." ***BMJ Global Health*** 6 (10): e006460.
5. Worksheets in .pdf and .doc formats are available at www.WISEscales.org. Files are also available at https://doi.org/10.21985/n2-xsw5-mz63.

Supplementary Text 2. Cognitive interview guide.

Read the introduction about the intervals for response, then the first HWISE item. All questions should be read pleasantly, exactly as they were written on the survey.

1. First write down their **answer** on the worksheet (in dropbox).

    - If the Participant Replies... "I don't know" "I still don't know" or "Not Applicable", then **repeat** the question. You could ask "Could you give me your best estimate?". Sometimes people say "I don't know" because they are taking time to think and want to gain time, they don't want to answer because of personal reasons, or actually does not know and has no opinion.

    - If they say not applicable, ask **why it is not applicable**.

2. Then ask the respondent to **rephrase** the question in their own words to be sure that the question was understood. Note down any difficulties.

    - One way to say it is: "Please tell me what I was asking in your own words."
    - Note if it was correct rephrased or not. If it was incorrect, note what the misunderstanding was.
    - If incorrect, explain to them what the difference between what the correct interpretation was and what they understood it to be. Ask them how the item could be phrased in a way that is easier to understand.

3. Then request the respondent to explain **how she came to the answer**.

    - Frequency. For example, if they say it happened "sometimes" ask them how they counted the months. You could also ask them if it is easier to think about if "About half the year" OR "Some but not every month" is easier.

    - Examples of the experience. Sometimes these prompts help people to explain what they said:

      "What were you thinking of when you answered that way?"
      "What made you say that?"
      "Why did you respond that way?"
      "Can you take me through the steps of how you came to that answer?"

Water Insecurity Scale – Proposed Questions

(READ:) Next, I will ask you about your experiences with water. For each experience, we want to know in how many months it happened to you during the LAST 12 MONTHS. Even if it happened once during a month, we'd like you to count that month. **(Read items)**

INTERVIEWER: Repeat scale as necessary after the first time. If respondent says, "in every month" code as "in almost every month."

	Never	In 1 or 2 months	In some but not every month	In almost every month	(DK)	(RF)
A) How often did you worry that you would not have enough water for all of your needs? Never, in 1 or 2 months, in some but not every month, or in almost every month?	**1**	**2**	**3**	**4**	**8**	**9**
B) Please think about where you get most of your water, such as a tap, well, borehole, bottled water, river, or stream. How often was this water source interrupted or limited in any way during the last 12 months?	**1**	**2**	**3**	**4**	**8**	**9**
C) How often could your clothes NOT be washed because of problems with water?	**1**	**2**	**3**	**4**	**8**	**9**
D) How often did you have to change schedules or plans because of problems with water?	**1**	**2**	**3**	**4**	**8**	**9**
E) Still thinking about the last 12 months, how often did you change what you ate because of problems with water?	**1**	**2**	**3**	**4**	**8**	**9**
F) How often were you NOT able to wash your hands after dirty activities because of problems with water?	**1**	**2**	**3**	**4**	**8**	**9**
G) How often were you NOT able to wash your body because of problems with water?	**1**	**2**	**3**	**4**	**8**	**9**
H) How often did you NOT have as much water to drink as you would have liked?	**1**	**2**	**3**	**4**	**8**	**9**
I) Still thinking about the last 12 months, how often did you feel angry because of problems you were experiencing with water?	**1**	**2**	**3**	**4**	**8**	**9**
J) How often did you go to sleep thirsty because there was not water to drink?	**1**	**2**	**3**	**4**	**8**	**9**
K) How often did you have NO useable or drinkable water whatsoever?	**1**	**2**	**3**	**4**	**8**	**9**
L) How often did you feel shame because of problems you were experiencing with water during the last 12 months?	**1**	**2**	**3**	**4**	**8**	**9**

Question M. In the last 12 months, how often did you drink or use water that you didn't want to because you had no other water? (Reasons for not wanting to drink, cook or use the water could be that you think it is unsafe or unpleasant tasting.)

1 2 3 4 8 9

4. Is there anything else I should know about problems with availability, access, use or reliability of water?

Sociodemographics

At the end of the interview, please note the respondent’s gender, age, ethnicity, language spoken, employment (if any), number of children in household, urbanicity, main water source(s), and any other characteristics that may be relevant to how they answered. For example, such characteristics may include if they are pregnant or have an injury that prevents them from accessing water.

Supplementary Tables 3-9. Pairwise assessment of measurement invariance of Individual Water Insecurity Experiences data, comparing two high-income countries (Australia and USA) to three low- and middle-income countries (Bangladesh, Brazil, and Uganda) in the Gallup World Poll (n=4,928).

Table 3. USA & Bangladesh*

	Configural invariance	Scalar invariance
**RMSEA (<0.06)**	0.046	0.048
**Upper RMSEA 90% CI (<0.06)**	0.052	0.053
**CFI (>0.95)**	0.997	0.995
**TLI (>0.95)**	0.996	0.996
**SRMR (<0.08)**	0.035	0.038

Table 4. USA & Brazil*

	Configural invariance	Scalar invariance
**RMSEA (<0.06)**	0.055	0.061
**Upper RMSEA 90% CI (<0.06)**	0.060	0.066
**CFI (>0.95)**	0.983	0.973
**TLI (>0.95)**	0.980	0.975
**SRMR (<0.08)**	0.048	0.053

Table 5. USA & Uganda*

	Configural invariance	Scalar invariance
**RMSEA (<0.06)**	0.060	0.051
**Upper RMSEA 90% CI (<0.06)**	0.065	0.056
**CFI (>0.95)**	0.980	0.981
**TLI (>0.95)**	0.976	0.982
**SRMR (<0.08)**	0.039	0.043

Table 6. Australia & Bangladesh*

	Configural invariance	Scalar invariance
**RMSEA (<0.06)**	0.040	0.037
**Upper RMSEA 90% CI (<0.06)**	0.046	0.042
**CFI (>0.95)**	0.998	0.997
**TLI (>0.95)**	0.997	0.998
**SRMR (<0.08)**	0.050	0.049

Table 7. Australia & Brazil*

	Configural invariance	Scalar invariance
**RMSEA (<0.06)**	0.047	0.049
**Upper RMSEA 90% CI (<0.06)**	0.052	0.053
**CFI (>0.95)**	0.986	0.981
**TLI (>0.95)**	0.983	0.982
**SRMR (<0.08)**	0.060	0.069

Table 8. Australia & Uganda*		
	**Configural invariance**	**Scalar invariance**
**RMSEA (<0.06)**	0.050	0.043
**Upper RMSEA 90% CI (<0.06)**	0.055	0.048
**CFI (>0.95)**	0.984	0.985
**TLI (>0.95)**	0.981	0.986
**SRMR (<0.08)**	0.053	0.062

Table 9. USA & Australia*		
	**Configural invariance**	**Scalar invariance**
**RMSEA (<0.06)**	0.037	0.029
**Upper RMSEA 90% CI (<0.06)**	0.043	0.034
**CFI (>0.95)**	0.988	0.990
**TLI (>0.95)**	0.985	0.991
**SRMR (<0.08)**	0.054	0.060

*Values in parentheses indicate suggested parameters; red shading indicates estimates outside the parameter.
RMSEA: root mean square error of approximation; CI: confidence interval; CFI: comparative fit index; TLI: Tucker-Lewis index; SRMR: standardized root mean square residual

Supplementary Table 10. Individual Water Insecurity Experiences Scale scores were associated with lower wealth and household size in US and Australia (Gallup World Poll 2022, n=1,992).[a]

	IWISE score		
	Coefficient	95% CI	*p*
**Wealth quintile** (ref: high)			
Mid-high	0.13	(-0.14, 0.39)	0.035
Middle	0.37	(0.04, 0.70)	0.026
Mid-low	0.51	(0.17, 0.84)	0.003
Low	1.11	(0.58, 1.63)	<0.001
**Difficult getting by on income** (ref: getting by on income)	2.21	(1.55, 2.87)	<0.001
**Number of household members**	0.13	(0.03, 0.23)	0.013

[a] Each model includes the exposure of interest and adjusts for site and the complex sampling strategy (to ensure proper standard errors).
CI: confidence interval
